# Curcumin Chemosensitizes 5-Fluorouracil Resistant MMR-Deficient Human Colon Cancer Cells in High Density Cultures

**DOI:** 10.1371/journal.pone.0085397

**Published:** 2014-01-03

**Authors:** Mehdi Shakibaei, Constanze Buhrmann, Patricia Kraehe, Parviz Shayan, Cora Lueders, Ajay Goel

**Affiliations:** 1 Institute of Anatomy, Ludwig-Maximilian-University Munich, Germany; 2 Investigating Institute of Molecular Biological System Transfer, Tehran, Iran; 3 Department of Thoracic and Cardiovascular Surgery, Laboratory for Tissue Engineering, German Heart Institute Berlin, Berlin, Germany; 4 Gastrointestinal Cancer Research Laboratory, Division of Gastroenterology, Baylor Research Institute and Charles A. Sammons Cancer Center, Baylor University Medical Center, Dallas, Texas, United States of America; Yong Loo Lin School of Medicine, National University of Singapore, Singapore

## Abstract

**Objective:**

Treatment of colorectal cancer (CRC) remains a clinical challenge, as more than 15% of patients are resistant to 5-Fluorouracil (5-FU)-based chemotherapeutic regimens, and tumor recurrence rates can be as high as 50–60%. Cancer stem cells (CSC) are capable of surviving conventional chemotherapies that permits regeneration of original tumors. Therefore, we investigated the effectiveness of 5-FU and plant polyphenol (curcumin) in context of DNA mismatch repair (MMR) status and CSC activity in 3D cultures of CRC cells.

**Methods:**

High density 3D cultures of CRC cell lines HCT116, HCT116+ch3 (complemented with chromosome 3) and their corresponding isogenic 5-FU-chemo-resistant derivative clones (HCT116R, HCT116+ch3R) were treated with 5-FU either without or with curcumin in time- and dose-dependent assays.

**Results:**

Pre-treatment with curcumin significantly enhanced the effect of 5-FU on HCT116R and HCR116+ch3R cells, in contrast to 5-FU alone as evidenced by increased disintegration of colonospheres, enhanced apoptosis and by inhibiting their growth. Curcumin and/or 5-FU strongly affected MMR-deficient CRC cells in high density cultures, however MMR-proficient CRC cells were more sensitive. These effects of curcumin in enhancing chemosensitivity to 5-FU were further supported by its ability to effectively suppress CSC pools as evidenced by decreased number of CSC marker positive cells, highlighting the suitability of this 3D culture model for evaluating CSC marker expression in a close to *vivo* setting.

**Conclusion:**

Our results illustrate novel and previously unrecognized effects of curcumin in enhancing chemosensitization to 5-FU-based chemotherapy on DNA MMR-deficient and their chemo-resistant counterparts by targeting the CSC sub-population. (246 words in abstract).

## Introduction

Colorectal cancer (CRC) is the third most frequent cancer affecting men and women equally worldwide [1]. Current therapies for the treatment of colorectal cancer mainly comprise 5-Fluorouracil-based chemotherapies that are used individually or in combination with oxaliplatin (FOLFOX) or anti-angiogenic agents, and/or anti-epidermal growth factor agents [2]. Although colon cancer incidence rates have declined somewhat, current therapies are associated with significant side effects, high expense and recurrence rates upwards of 50%, primarily due to the development of acquired chemoresistance to conventional chemotherapeutics [3], [4]. These limitations highlight the imperative and urgent need for identifying and developing novel and safe treatment strategies that can help overcome chemoresistance and enhance tumor cell response to anti-tumor drugs.

Carcinogenesis is believed to be a multistep process that results from a stepwise accumulation of genetic alterations in various genes (e. g. metastasis-associated genes, oncogenes, tumor suppressor genes) leading to progressive conversion of healthy cells to tumor cells [5], [6]. It is now further recognized, that epigenetic alterations such as aberrant DNA methylation, histone modifications, chromosome remodeling and damage to the mismatch repair (MMR) system, also markedly influence CRC development, [5], [7]. Damage to the MMR system causes genetic instability as it is important for proof reading DNA synthesis errors during replication, leading to altered cell phenotypes, enhanced susceptibility for neoplastic transformation and facilitating development of chemo-resistant cells [8], [9].

During tumorigenesis and tumor dissemination including colon cancer, cancer cells require self-renewal capability, similar to that exhibited by stem cells. It is now widely accepted that cancer pathogenesis in most tumors, including CRC, is driven by a subset of tumor cells that exhibit stem cell characteristics similar to physiologic stem cells, including self-renewal abilities and pluripotency [10], [11] and that these cancer stem cells (CSC) have the potential to invade and form distant metastasis [12], [13], [14]. In the colon, these colonic CSC aberrantly differentiate generating a bulk of tumor cells with the larger fraction composed of more differentiated cells and a small fraction of stem cells, which eventually replace the healthy colonic stem cells and the entire colonic crypt is colonized by cancer stem cells and their progeny [10]. A set of specific markers have been identified for colonic CSC, including CD133^+^, CD 44^+^, CD166^+^ and ALDH1^+^ [15], [16]. Relapse of tumors after apparently successful chemotherapy is believed to be by virtue of chemo-resistant CSCs that evade death by chemotherapeutic drugs [17]. Therefore, new therapeutic agents that can successfully target CSCs, is very likely the most promising therapeutic strategy in meeting this tremendous clinical challenge.

Emerging literature suggests that many dietary components can directly or indirectly regulate inflammatory responses in the bowel by modulating the intestinal barrier function [18]. Furthermore, several naturally occurring dietary compounds have been shown as anti-cancer therapeutic agents [19], [20], [21], [22]. Indeed evidence is emerging that conventional chemotherapy in CRC significantly benefits through combinational treatments with some of such naturally occurring dietary polyphenols [5], [23], [24]. One such botanical, curcumin (diferuloylmethane), a yellow spice derived from the rhizomes of *curcuma longa*, has a long tradition as an anti-inflammatory agent in the traditional Indian Ayurvedic system of medicine. In addition, several studies have shown that curcumin acts as a potent chemo- and radiosensitizer in multiple human cancers [25], [26] and can inhibit cell growth of MMR system deficient colon cancer cells [27], [28]. In a previous study, we demonstrated that curcumin enhanced the chemosensitivity of 5-FU in colorectal cancer cell lines by targeting NF-κB, Src and NF-κB-dependent regulated gene products [5]. Furthermore it has been shown that targeting colon cancer cells with 5-FU and oxaliplatin (FOLFOX) in combination with curcumin attenuated EGF-R, IGF-1R and Akt signaling pathway and markedly reduced cancer stem cell marker positive cells and caused disintegration of colonospheres [23], [24].

We have recently shown that combined treatment of curcumin with 5-FU induces more significant cytotoxicity in DNA MMR-deficient CRC monolayer cultures, compared to each agent individually [5]. Accordingly, the aim of the present study was to investigate whether curcumin alone or in combination with 5-FU could affect DNA MMR-deficient colon cancer cells that are inherently resistant to 5-FU in modulating colonosphere formation and CSC activity in 3D cultures.

## Materials and Methods

### Cell Lines and Cell Culture

Human colon cancer cells (HCT116; MMR-deficient) were obtained from European Collection of Cell Cultures (Salisbury, UK). HCT116+ch3, is a MMR-proficient cell line, which was created in our laboratory by the stable transfection of chromosome 3 bearing a wild-type copy of the *hMLH1* gene, as described previously [29]. We also generated 5-FU resistant derivatives of these cell lines, referred to as HCT116R and HCT116+ch3R respectively, that were created by repetitive treatment of the parental cell lines to increasing concentrations of 5-FU over a 10–12 month period. Both the parental and 5-FU resistant cell lines were used to investigate the efficacy of individual and combined 5-FU and curcumin treatments. The cells were maintained in tissue culture flasks in Dulbecco’s modified Eagle medium (DMEM; 4.5 g/L D-glucose) supplemented with 10% FBS and 1% antibiotic/antimycotic in a humidified incubator at 37°C in an atmosphere of 95% air and 5% CO_2_. The medium was changed every three days, and cells were passaged using Trypsin/EDTA.

### Antibodies

Monoclonal antibodies to ALDH1 were purchased from Acris Antibodies GmbH (Herold, Germany). Monoclonal antibodies to CD133 and CD44 were purchased from Abcam PLC (Cambridge, UK). Antibodies to β-actin (A5316) were from Santa Cruz Biotechnology (Santa Cruz, CA). Monoclonal anti-PARP [poly(ADP-ribose)polymerase] (7D3-6) antibody was purchased from Becton Dickinson (Heidelberg, Germany). Alkaline phosphatase linked sheep anti-mouse and sheep anti-rabbit secondary antibodies for immunoblotting were purchased from Millipore (Schwalbach, Germany). All antibodies were used at concentrations and dilutions recommended by the manufacturer.

### Growth Media, Chemicals, and Cytokines

Growth medium (Ham’s F-12/Dulbecco’s modified Eagle’s medium (50∶50) containing 10% fetal bovine serum (FBS), 25 µg/ml ascorbic acid, 50 IU/ml streptomycin, 50 IU/ml penicillin, 2.5 µg/ml amphotericin B, essential amino acids and L-glutamine) was obtained from Seromed (Munich, Germany). Trypsin/EDTA (EC 3.4.21.4) was purchased from Biochrom (Berlin, Germany). Epon was obtained from Plano (Marburg, Germany). 5-FU was purchased from Sigma (Munich, Germany). Curcumin (BCM-95) was a generous gift from Dolcas Biotech (Landing, NJ, USA). Curcumin was prepared by dissolving it in dimethylsulfoxide (DMSO) at a stock concentration of 5000 µM and stored at −20°C. Serial dilutions were prepared in culture medium.

A 100 mM stock of 5-FU was prepared in absolute DMSO and stored at −20°C. The concentration of DMSO was less than 1% of drug treatment. For treatment, 5-FU was diluted in DMEM and added to cultures to give the desired final concentration. After 70–80% confluency, the cells were treated with 5-FU or curcumin individually, or their combination.

### Cell Viability Assay

The cell viability was evaluated by the 3-(4,5-dimethylthiazol-2-yl)-2,5-diphenyltetrazolium bromide (MTT) uptake method as described previously [30]. Briefly, HCT116, HCT116+ch3 cells and the 5-FU chemo-resistant cell lines were seeded in a 96-well plate (1000 per well) and exposed to different concentrations of individual 5-FU or curcumin in triplicate for the indicated time periods to obtain the IC_50_ values (50% cell growth inhibitory concentrations). Additionally, in another set of experiments, cells were pretreated with 5 µM curcumin for 12 h and then co-treated with different concentrations of 5-FU (0, 0.1, 1, 2, 3, 4 µM) for 24 h to obtain the optimum dose for combination treatment. Cells were washed twice with PBS and subsequently, MTT solution (5 mg/mL) was added to each well and the plate was incubated for 2 h at 37°C. The lysis buffer (20% SDS and 50% dimethyl formamide) was added, and the cells were incubated overnight at 37°C. The absorbance of the cell suspension was measured at 570 nm (OD570) using Revelation 96-well multiscanner plate reader (Bio-Rad Laboratories Inc. Munich, Germany). The data obtained were calculated and were represented as percentage of survival relative to controls. This experiment was repeated 3 times independently, and statistical analysis was done to obtain the final values.

### Formation and Inhibition of Colonosphere Colonies

For tumor colonosphere formation assay, 10 µl drop, approximately 2 million cells, HCT116, HCT116+ch3 and their respective 5-FU chemo-resistant derivative cell lines were plated on a cellulose filter on top of a steel mesh bridge [31]. Cell culture medium reached the filter medium interface and cells were nurtured through diffusion. This model allows the cells to aggregate, and form a distinct pellet, which was examined after 1–10 days. The HCT116, HCT116+ch3 and their respective 5-FU chemo-resistant cell lines were treated with curcumin (20 µM), 5-FU (5 µM) or their combination (curcumin 5 µM and 5-FU 0.1 µM) for 1, 3, 7 and 10 days, respectively. Applied concentrations were calculated and represented as percentage survival with respect to untreated controls. The IC_50_ was defined as the drug concentration required to inhibit HCT116, HCT116R or HCT116+ch3 and HCT116+ch3R by 50% relative to controls. IC_50_ values were estimated from the dose response curve. Data were derived from at least three independent experiments. Colonosphere formation was assessed by light microscopy, DAPI (4′,6-Diamidino-2-phenylindole, Sigma) and electron microscopy.

### Transmission Electron Microscopy (TEM)

Electron microscopy was performed as previously described [32]. Briefly, high density cell cultures, treated as described above, were fixed for 1 h in Karnovsky’s fixative followed by post-fixation in 1% OsO_4_ solution. After dehydration in an ascending alcohol series, cultures were embedded in Epon and cut ultrathin with a Reichert-Jung Ultracut E (Darmstadt, Germany). Sections were contrasted with a mixture of 2% uranyl acetate/lead citrate and examined with a transmission electron microscope (TEM 10, Zeiss, Institute for pharmacology Berlin, Germany).

### Quantification of Mitochondrial Changes (MC) and Apoptotic Cell Death

Ultrathin sections of the samples were prepared and evaluated with an electron microscope (TEM 10; Zeiss, Institute for pharmacology Berlin, Germany). To quantify the MC and apoptotic cells, the number of cells with morphological features of apoptotic cell death was determined by scoring 100 cells from 25 different microscopic fields.

### DAPI Staining for Apoptotic Cells

Chromatin condensation and apoptotic cells were examined by nuclear staining with DAPI (4′,6′-Diamidino-2-phenylindole, Sigma), as previously described [32]. Briefly, the high density cultures were immersed in O.C.T. embedding medium and immediately frozen in liquid nitrogen. Eight to ten µm thick sections were cut. Sections were fixed with methanol for 30 min at 4°C in the dark. Subsequently, the cultures were washed twice with PBS, and then 1 µl of DAPI solution (5 mg/ml) in five ml PBS was spread over the cultures followed by incubation for 20 min in the dark. Labelled cells were washed repeatedly with PBS to remove the excess DAPI stain, covered with fluoromount mountant and evaluated under a fluorescence microscope (Leica, Germany). Experiments and analysis were performed in triplicate.

### Western Blot Analysis

To determine the effect of curcumin, 5-FU or curcumin/5-FU on cleavage of PARP and the expression of cancer stem cell (CSC) markers, whole cell lysates were prepared and fractioned by SDS-PAGE [33]. Briefly, cells were rinsed in PBS, and the proteins were extracted with lysis buffer (50 mM Tris/HCl (pH 7.2), 150 mM NaCl, 1% (v/v) Triton X-100, 1 mM sodium orthovanadate, 50 mM sodium pyrophosphate, 100 mM sodium fluoride, 0.01% (v/v) aprotinin, pepstatin A (4 µg/ml), leupeptin (10 µg/ml), and 1 mM phenylmethylsulfonyl fluoride (PMSF)) for 30 min on ice. After adjusting the total protein concentration, equal quantities (500 µg protein per lane) of total proteins were separated by SDS-PAGE (7.5% or 12% gels) under reducing conditions. Separated proteins were transferred to nitrocellulose membranes and incubated in blocking buffer (5% (w/v) skimmed milk powder in PBS, 0.1% Tween 20) for 1 h at room temperature. Membranes were incubated overnight with the primary antibody diluted in blocking buffer at 4°C on a shaker, washed 3 times with blocking buffer, and then incubated with the secondary antibody conjugated with alkaline phosphatase for 90 min at room temperature. Membranes were washed 3 times in 0.1 M Tris (pH 9.5) containing 0.05 M MgCl_2_ and 0.1 M NaCl. Finally, specific antigen-antibody complexes were detected using nitroblue tetrazolium and 5-bromo-4-chloro-3-indoylphosphate (*p*-toluidine salt; Pierce, Rockford, IL, USA) as substrates for alkaline phosphatase.

### Statistical Analysis

Numerical data are expressed as the mean values (+/−SD) for a representative experiment performed in triplicate. The means were compared using Student’s *t* test assuming equal variances. Differences were considered to be statistically significant if the *p* value was less than 0.05.

## Results

Human MMR-deficient and -proficient CRC cells (HCT116, HCT116+ch3) and their respective 5-FU chemo-resistant derivative cell lines (HCT116R, HCT116+ch3R) were used in our study to investigate the effect of curcumin or/and 5-FU on cell viability, apoptosis and cancer stem cells (CSC) in high density cultures.

### MMR- Deficient CRC Cells and Their Respective 5-FU Resistant Cells are Sensitive to Curcumin

**Figure 1:**
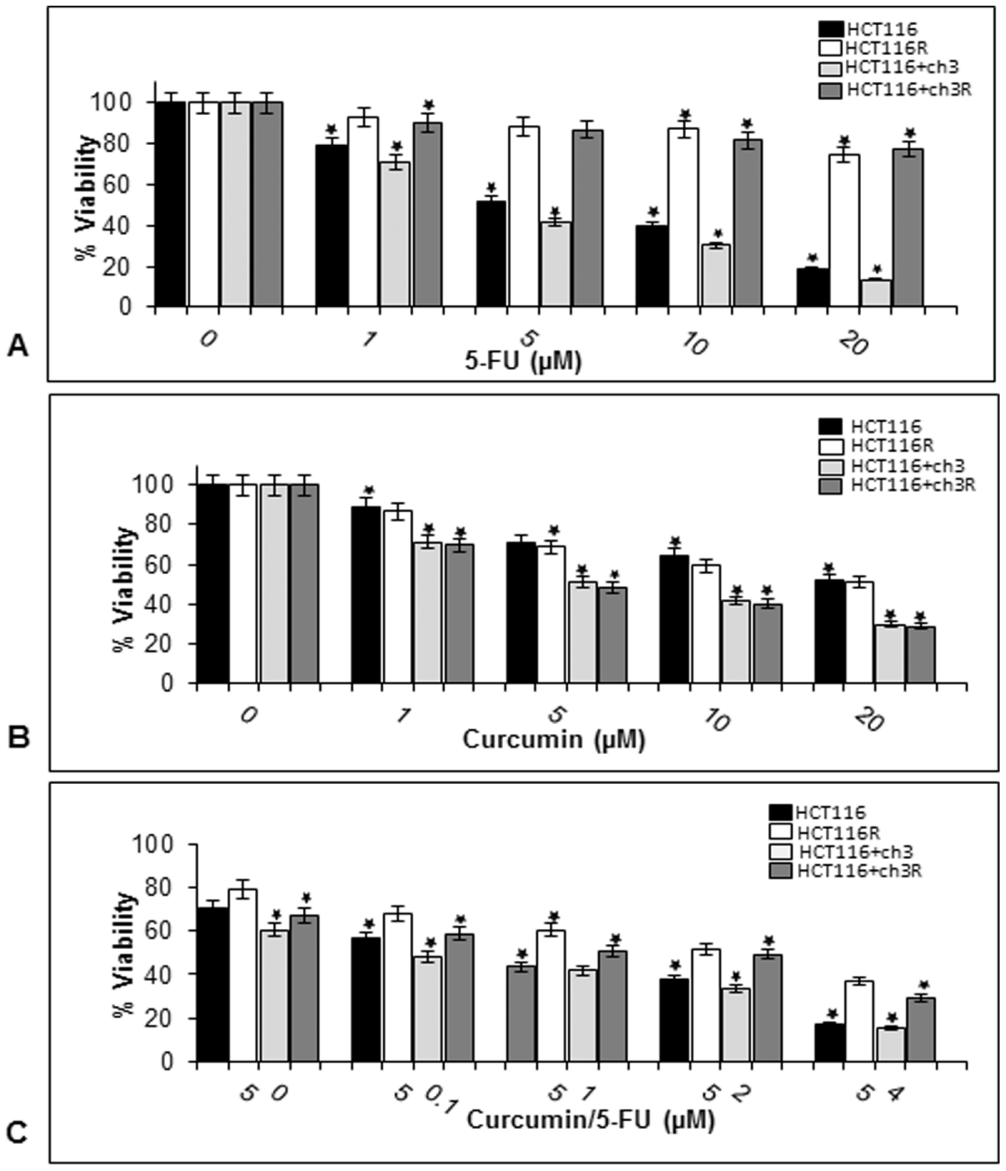
Cell viability is reduced by 5-FU, curcumin and the combination treatment in HCT116, HCT116+ch3 and the corresponding 5-FU resistant cell lines. HCT116, HCT116+ch3, HCT116R and HCT116+ch3R cell lines were treated with different concentrations of 5-FU (0, 1, 5, 10 and 20 µM) alone (A) for 24 hours, different concentrations of curcumin (0, 1, 5, 10 and 20 µM) alone (B) for 24 hours, or were pre-treated with curcumin 5 µM for 4 h, and then exposed to different concentrations of 5-FU (0, 0.1, 1, 2, 4) for 24 hours (C). Cell viability was measured with the MTT method and IC_50_ determined at 50% growth inhibition. The results are provided as mean values with standard deviations from at least three independent experiments. Values were compared to the control and statistically significant values with p<0.05. Significant values are marked with (*).

The cytotoxic effects of 5-FU or curcumin on four colon cancer cell lines were determined using the MTT assay. The cells were treated with different concentrations of 5-FU (0, 1, 5, 10 and 20 µM) or curcumin (0, 1, 5, 10 and 20 µM) and cell viability was examined by MTT assay (Fig. 1A & 1B). We observed that 5-FU blocked the proliferation of the cell lines HCT116, HCT116+ch3 in a dose-dependent manner with an IC_50_ value of 5 µM, whereas, their respective 5-FU resistant derivatives did not have any response to 5-FU treatment (Fig. 1A). The HCT116+ch3 cells and the corresponding 5-FU chemo-resistant cell lines were equally sensitive to curcumin (IC_50_ 5 µM), whereas the DNA MMR-deficient cell lines HCT116 and HCT116R cells were less sensitive to curcumin compared to the DNA MMR-proficient cell lines HCT116+ch3 and HCT116+ch3R (IC_50_ 20 µM; Fig. 1B). These results suggest that the restoration of hMLH1 activity in the HCT116 cell line, by introduction of chromosome 3, was associated with an increased sensitivity to 5-FU.

To evaluate cell sensitivity to the combination of curcumin and 5-FU treatment, we pretreated HCT116, HCT116+ch3 and the respective 5-FU chemo-resistant cells first with 5 µM curcumin followed by treatment with different concentrations of 5-FU (0, 0.1, 1, 2 and 4 µM) for 24 h (Fig. 1C). MTT assays were performed and IC_50_ values were determined. The results show that curcumin significantly enhanced the cytotoxicity of 5-FU for both cell lines (HCT116 and HCT116+ch3) at approximately 0.1 µM 5-FU (Fig. 1C). Interestingly, pretreatment with 5 µM curcumin reduced IC_50_ values for 5-FU to 2 µM in the 5-FU resistant HCT116R and HCT116+ch3R cells lines (p<0.05; Fig. 1C). These results suggest that DNA MMR-deficient HCT116R and DNA MMR-proficient HCT116+ch3R cells pretreated with curcumin were more sensitive to 5-FU than cells treated with 5-FU alone and the introduction of chromosome 3 in HCT116 cells showed an increased sensitivity of the cells to the treatment with 5-FU and/or curcumin compared to the HCT116 cells.

### Curcumin and/or 5-FU Suppress Colonosphere Growth of MMR-deficient CRC Cells and Their Respective 5-FU Resistant Cells in High Density Cultures

**Figure 2:**
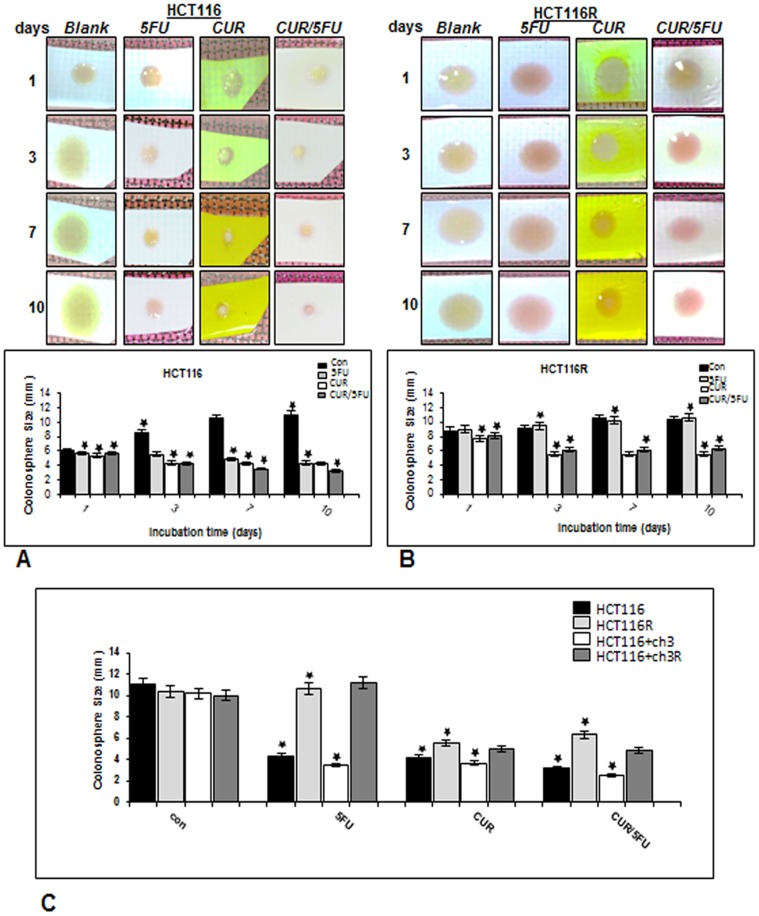
Curcumin inhibits colonosphere formation in HCT116, HCT116+ch3 and their respective 5-FU-chemoresistant cell lines by heightening the chemosensitivity to 5-FU treatment in high density cultures. High density cultures of HCT116 (A) or HCT116R (B) cells were either left untreated or were treated with 5-FU (5 µM), curcumin (20 µM), or 5-FU/curcumin in combination (0.1/5 µM). Cultures were evaluated after 1, 3, 7, and 10 days, and pictures of the native cultures taken. Pictures are representative of three individual experiments. Colonosphere size was measured and results presented are mean values with standard deviations from three independent experiments. C: High density cultures of HCT116, HCT116+ch3 and their respective 5-FU-chemoresistant cells were either left untreated or were treated with 5-FU (5 µM), curcumin (20 µM), or 5-FU/curcumin in combination (0.1/5 µM). Cultures were evaluated after 10 days, colonosphere size was measured and results presented are mean values with standard deviations from three independent experiments. Significant values are marked with (*).

To evaluate the effects of 5-FU and/or curcumin either alone or in combination on the size of colonospheres, a prominent feature of cancer stem cells [34], three-dimensional high-density cultures [31] of the HCT116 and the corresponding chemo-resistant cell lines were performed (Fig. 2A–C).

In untreated control cultures, the results showed that both HCT116 and HCT116R cells formed spheroid colonies, with a time-dependent increase in the size of colonospheres during the 10 day period (Fig. 2A, 2B). Treatment of HCT116 cultures with 5-FU alone, in contrast to their corresponding 5-FU resistant HCT116R cell line, dramatically reduced the size of colonospheres compared with the corresponding controls (Fig. 2A, 2B). As expected, treatment of 5-FU resistant cells with 5-FU individually did not have a negative effect on colonosphere size (Fig. 2B). In contrast, in cultures treated with curcumin alone and/or 5-FU, the colonosphere size was significantly decreased when compared with the corresponding controls (Fig. 2A, 2B, 2C). The treatment with curcumin alone or the combination treatment was shown to be highly effective in inhibiting the sphere forming ability of all four CRC cell lines. The sizes of colonospheres in with curcumin treated groups were significantly smaller compared to the control groups, indicating that a) curcumin by itself supressed colonosphere size in DNA MMR deficient HCT 116 cells, and b) curcumin sensitized HCT116R and HCT116+ch3R cells to 5-FU-induced cytotoxicity (Fig. 2A, 2B, 2C). At the end of the experimental period (10 days), it could be observed that while the control cells formed well-developed colonospheres, the 5-FU resistant CRC cells exposed to curcumin alone or in combination treatment showed significantly smaller spheroid formation (Fig. 2C). Taken together, these results indicate that colonoshere size was markedly decreased in high density cultures treated either with curcumin alone or combination of curcumin and 5-FU, indicating a colonosphere inhibiting effect of curcumin and/or 5-FUon HCT116, HCT116+ch3 and their respective 5-FU resistant derivatives.

### Curcumin and/or 5-FU Increase Cytotoxicity of Colonospheres of MMR-deficient CRC Cells and Their Respective 5-FU Resistant Cells in High Density Cultures

**Figure 3:**
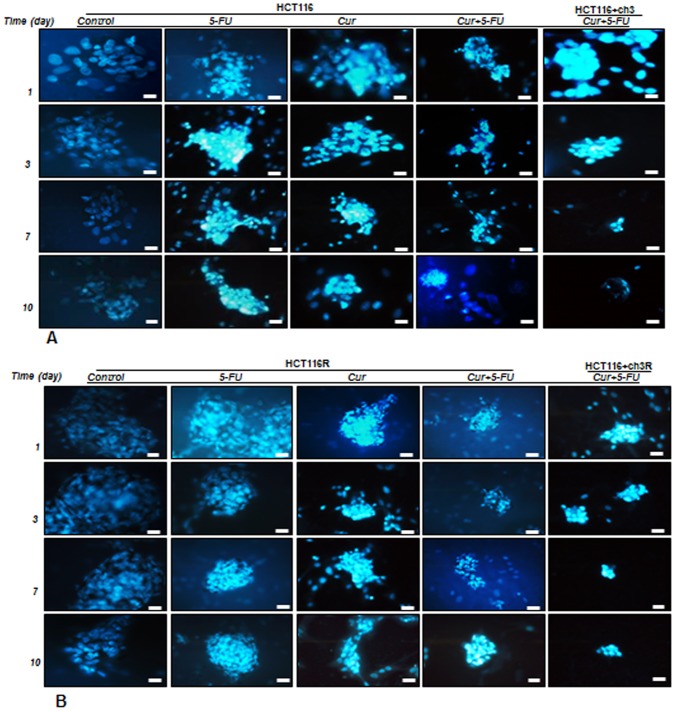
Cytotoxicity of 5-FU, curcumin and the combination treatment on colon cancer cells in high density cultures. High density cultures of HCT116, HCT116+ch3 (A), or HCT116R and HCT116+ch3R (B) were either left untreated or were treated with 5-FU (5 µM), curcumin (20 µM), or 5-FU/curcumin in combination (0.1/5 µM). Cultures were evaluated after 1, 3, 7, and 10 days, and stained with Hoechst 33258 (DAPI) to reveal apoptotic changes of the cell nuclei. Pictures are representative of three individual experiments.

To examine whether the colonosphere formation-inhibitory effect of curcumin and 5-FU in HCT116, HCT116+ch3, HCT116R and HCT116+ch3R cell lines is related to the induction of apoptosis, treated cells were evaluated using DAPI staining, which showed that apoptotic bodies containing nuclear fragments and chromatin condensation were generated within the apoptotic pool of cells (Fig. 3A, 3B). Indeed, incubation of HCT116 and HCT116R cells in serum-starved medium resulted in the formation of colonosphere during a period of 10 days. However, incubation of HCT116 and HCT116R cells with 5-FU, curcumin, or curcumin together with 5-FU for 10 days resulted in a marked disintegration of colonosphere(s) when compared with their corresponding controls (Fig. 3A, 3B). Highest disintegration was observed in response to the combination of curcumin and 5-FU in HCT116+ch3 and HCT116+ch3R cell lines (Fig. 3A, 3B).

### Curcumin Enhances 5-FU-mediated Apoptosis of MMR-deficient CRC Cells and Their Respective 5-FU Resistant Cells in High Density Cultures

**Figure 4:**
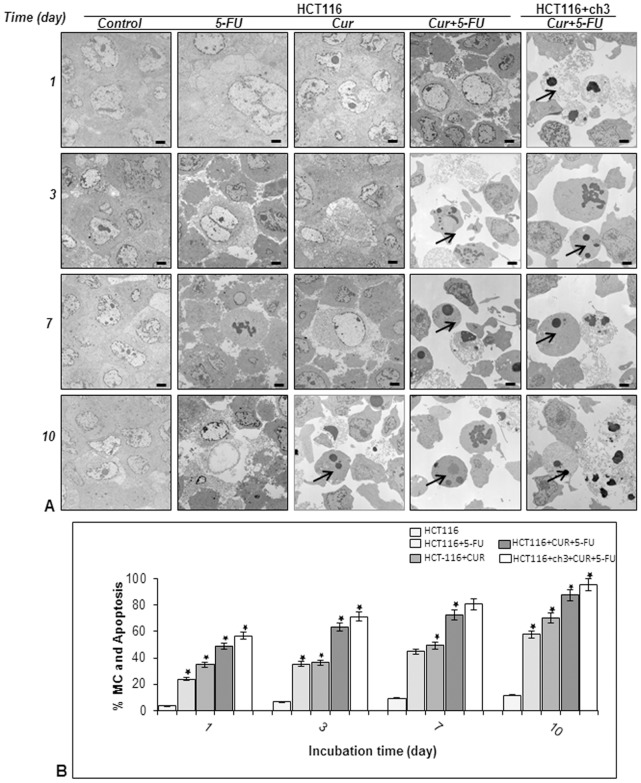
Ultrastructural evaluation of cytotoxicity of 5-FU, curcumin and the combinational treatment on HCT116 and HCT116+ch3 cells in high density cultures. A: High density cultures of HCT116 and HCT116+ch3 were either left untreated or were treated with 5-FU (5 µM), curcumin (20 µM), or 5-FU/curcumin in combination (0.1/5 µM). Cultures were evaluated after 1, 3, 7, and 10 days, and evaluated ultrastructurally with a transmission electron microscope. At the earliest time point when apoptosis (arrows) was first detected, images are highlighted in red boxes. Micrographs shown are representative of three individual experiments. Magnification: x5000, bar = 1 µm. B: Mitochondrial changes (MC) and apoptosis were quantified by counting 100 cells with morphological features of apoptotic cell death from 25 different microscopic fields and results presented are mean values with standard deviations from three independent experiments. Significant values are marked with (*).

**Figure 5:**
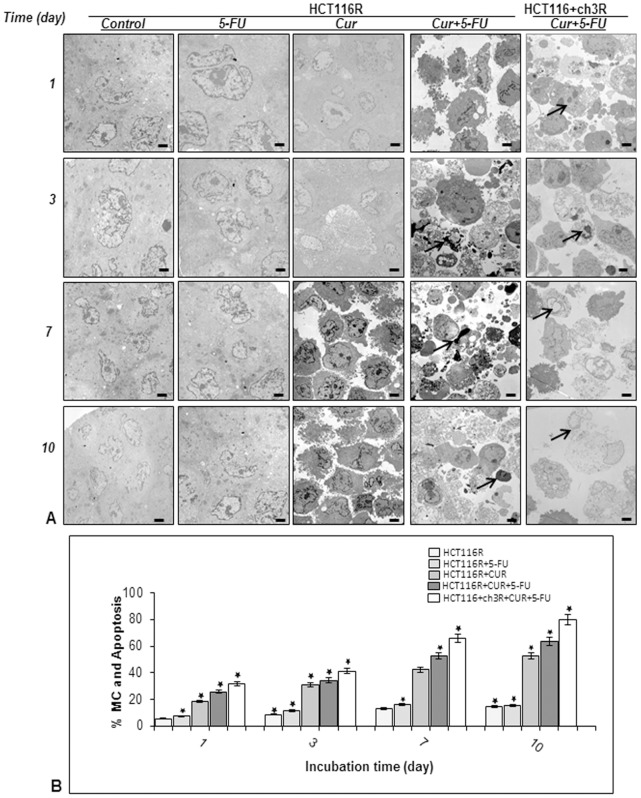
Ultrastructural evaluation of cytotoxicity of 5-FU, curcumin and the combinational treatment on HCT116 5-FU-chemoresistant and HCT116+ch3 5-FU-chemoresistant cell lines in high density cultures. A: High density cultures of HCT116R and HCT116+ch3R were either left untreated or were treated with 5-FU (5 µM), curcumin (20 µM), or 5-FU/curcumin in combination (0.1/5 µM). Cultures were evaluated after 1, 3, 7, and 10 days, and evaluated ultrastructurally with an electron microscope. At the earliest time point when apoptosis (arrows) was first detected images are highlighted in red boxes. Micrographs shown are representative of three individual experiments. Magnification: x5000, bar = 1 µm. B: Mitochondrial changes (MC) and apoptosis were quantified by counting 100 cells with morphological features of apoptotic cell death from 25 different microscopic fields and results presented are mean values with standard deviations from three independent experiments. Significant values are marked with (*).

To examine whether the growth-inhibitory effect of curcumin and 5-FU in colonosphere formation in three-dimensional high-density cultures is related to the induction of apoptosis, ultrastructural evaluations were performed (Fig. 4 & 5) in HCT116, HCT116+ch3, HCT116R and HCT116+ch3R cells treated with 5-FU (5 µM) and curcumin (20 µM) individually, or 5-FU/curcumin in combination (0.1/5 µM) for 1, 3, 7, and 10 days. In control cultures, HCT116, HCT116+ch3 and their 5-FU-resistant counterpart cell lines exhibited large rounded viable cells (containing distinct distribution of endoplasmic reticulum, mitochondria and other cellular organelles) and these cells made intimate cell-to-cell contact (Fig. 4A, 5A). Treatment of HCT116 cells with 5-FU or curcumin alone resulted in degeneration of cell organelles, mitochondrial swelling and appearance of multiple vacuoles, with prominent signs of apoptosis especially in curcumin-treated high density cultures around day 10 (Fig. 4A–B). These included areas of condensed heterochromatin within the nuclei, and multiple autophagocytic cytoplasmic vacuoles. Similar observations were made for HCT116+ch3 cells (data not shown). In contrast, such effects could not be observed in 5-FU or curcumin treated HCT116R (Fig. 5A–B) or HCT116+ch3R cells (data not shown). However, a combinatorial treatment of 5-FU and curcumin over 10 days markedly enhanced degeneration of all tumor cells. Marked degenerative changes and apoptotic cells were detected around day 3 in HCT116 (Fig. 4A–B) and HCT116R cells (Fig. 5A–B), that were already visible around day 1 in HCT116+ch3 cells (Fig. 4A–B) and HCT116+ch3R cells (Fig. 5A–B). Quantification and statistical analysis of the ultrastructural data highlights the prominent effects of combined 5-FU and curcumin treatment on inducing and enhancing mitochondrial changes (MC) and apoptotic effects in HCT116 cells (Fig. 4B) and HCT116R cells (Fig. 5B).

### Curcumin Potentiates the Antitumor Effect of 5-FU through Apoptosis Pathway in HCT116, HCT116+ch3 Cells and Their Respective 5-FU Resistant Cells in High Density Cultures

**Figure 6:**
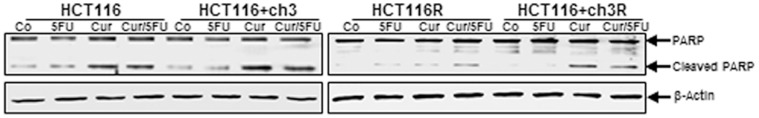
Effect of curcumin on 5-FU-induced apoptotic signaling in HCT116, HCT116+ch3 and their respective 5-FU-chemoresistant cell lines in high density cultures. High density cultures of HCT116 and HCT116+ch3 (left lanel) and of HCT116R and HCT116+ch3R (right panel) cells were either left untreated or were treated with 5-FU (5 µM), curcumin (20 µM), or 5-FU/curcumin in combination (0.1/5 µM). Cultures were evaluated after 3 days and whole cell lysates prepared and analyzed by western blotting for cleavage of PARP. Western blots shown are representative of three independent experiments. The housekeeping protein β-actin served as a positive loading control in all experiments.

Since ultrastructural evaluation with electron microscopy results showed that 5-FU+/−curcumin-induced apoptosis (Fig. 4–5), and PARP appears to be involved in the induction of apoptosis in tumor cells [35], therefore we investigated whether curcumin potentiates PARP cleavage in with 5-FU-treated HCT116, HCT116+ch3 and their corresponding 5-FU-resistant counterparts. As shown in Fig. 6, immunoblot analysis demonstrated that the cleavage of PARP was enhanced, when the cells were exposed to curcumin (20 mM) or 5-FU (5 mM) alone, or in the combination of curcumin and 5-FU (0.1/5 µM). These effects were more pronounced in combination treatment with curcumin and 5-FU than in single treatments (Fig. 6).

### Curcumin has Potent Chemosensitization Effect on Colon Cancer Stem Cells in MMR-Deficient and -proficient CRC Cells in High Density Cultures

**Figure 7:**
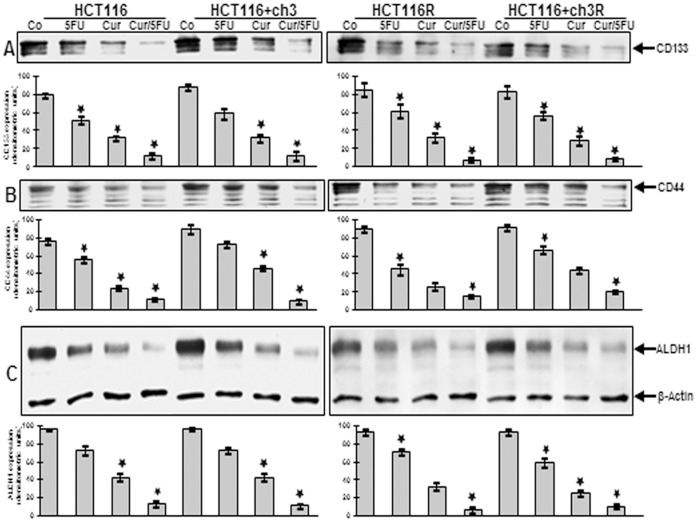
Colon cancer stem cell marker expression in high density cultures as shown by western blotting evaluation. High density cultures of HCT116 and HCT116+ch3 (left lanel, A, B, C) and of HCT116R and HCT116+ch3R (right panel, A, B, C) cells were either left untreated or were treated with 5-FU (5 µM), curcumin (20 µM), or 5-FU/curcumin in combination (0.1/5 µM). Cultures were evaluated after 3 days and whole cell lysates prepared and analyzed by western blotting and quantitative densitometry for expression of CD133, CD44 and ALDH1. Western blots shown are representative of three independent experiments. The housekeeping protein β-actin served as a positive loading control in all experiments. Significant values are marked with (*).

To demonstrate the chemosensitization effect of curcumin on colon CSC markers CD133, CD44 and ALDH1 expression in high density cultures, western blotting analysis was performed (Fig. 7). High density cultures of HCT116, HCT116+ch3 and their corresponding 5-FU-resistant counterparts were either left untreated, or were treated with 5-FU (5 µM) or curcumin (20 µM) alone, or 5-FU/curcumin in combination (0.1/5 µM) for 12 h. Control HCT116 and HCT116+ch3 high density cultures had strong colon CSC marker expression (Fig. 7A, B, C). In contrast, immunoblotting analysis of whole cell lysates showed marked down regulation of CD133, CD44 and ALDH1 in HCT116, HCT116+ch3, HCT116R and HCT116+ch3R cells in high density cultures treated with either 5-FU, curcumin or the combination of 5-FU and curcumin (Fig. 7A, B, C). The combination treatment of 5-FU/curcumin, although it contains only low concentrations of 5-FU compared to 5-FU treatment alone, proved to be most effective in down regulating colon stem cell marker expression. Densitometric analysis of typical Western blot experiments performed in triplicate show down regulation of CD133, CD44 and ALDH1 in HCT116, HCT116+ch3, HCT116R and HCT116+ch3R cells in high density cultures treated with either 5-FU, curcumin or the combination of 5-FU and curcumin (Fig. 7A, B, C). Taken together, this demonstrates the strong chemosensitizing effect of curcumin on colon CSC in both MMR-deficient and –proficient tumor cells in high density cultures.

## Discussion

The major purpose of this study was to investigate the efficacy of curcumin, a pharmacologically safe natural agent, for its potential chemopreventive and chemosensitization properties to 5-FU in 5-FU resistant mismatch repair deficient (MMR-deficient) colorectal cancer (CRC) cells in a three dimensional high density environment. We herein report that in high density culture curcumin alone or in combination with 5-FU significantly blocked proliferation, formation of colonospheres, induced apoptosis and down regulated colon cancer stem cell markers in MMR-deficient 5-FU resistant cells.

It has been reported that 5-FU is routinely used for the management of patients with CRC [36]. However, the use of 5-FU as a colorectal cancer chemotherapeutic agent has been somewhat limited due to the toxicities, limited success and associated adverse side effects. Recent research has suggested that plant polyphenols, such as curcumin, the major compound of turmeric and a nontoxic, naturally occurring dietary agent, might be used to sensitize tumor cells to chemotherapeutic therapy by suppression of pathways that lead to the development of chemoresistance in cancer cells [5], [23], [24], [37].

In this report, we observed increased sensitivity of HCT116+ch3 and their respective 5-FU chemo-resistant derivative cells to the treatment with 5-FU and/or curcumin compared to HCT116 and their respective 5-FU chemo-resistant derivative cells, suggests that these two cell populations respond differentially to 5-FU. Further, it has been previously reported that deficiencies in the MMR system increase the rate of genomic mutations and enhance the susceptibility to tumors including colorectal cancer [38], [39] and that damage to the MMR system through epigenetic alterations causes genetic instability and facilitates neoplastic transformation [8], [9]. Interestingly, it has been shown that inactivation of a MMR gene such as hMLH1, which can cause microsatellite instability (MSI), in colorectal cancer patients reduces 5-FU induced apoptosis [40]. Indeed, this observation is consistent with the reports of Sargent and colleagues that CRC patients with defective DNA mismatch repair (III stage) do not benefit from 5-FU treatment [41], suggesting that different mismatch colon cancer cells could exhibit different chemosensitivity patterns. Moreover, it has been reported that curcumin can inhibit cell growth of MMR-deficient colon cancer cells [27], [28]. Additionally Jiang et al. propose that anti-tumorigenic effect of curcumin is based on failure of MMR deficient tumor cells to develop curcumin induced double strand breaks and in the end this results in triggering apoptosis [42]. Therefore, we purposely chose the MMR-deficient colon carcinoma cell lines HCT116 and its counterpart 5-FU resistant derivative clones (HCT116R), together with an isogenic clone of HCT116 cells bearing a restored hMLH1 gene through a chromosome 3 transfer (HCT116+ch3) and its corresponding isogenic 5-FU resistant derivative clones (HCT116+ch3R). Our results demonstrate that with curcumin the proliferation of MMR-deficient and proficient CRC cells as well as their corresponding 5-FU resistant derivatives was suppressed and the effect of 5-FU was potentiated in a dose-dependent manner, thereby reducing the proliferation of CRC cells significantly as evidenced by the MTT assays and reduced colonosphere formation. This is in agreement with previous reports that curcumin exhibits synergistic activity with 5-FU against Tumor cells [24], [43], [44]. Recently we have already shown a synergistic, pro-apoptotic effect of curcumin and 5-FU in MMR-deficient and proficient colorectal cancer cells in monolayer cultures [5]. Novel in the present report, we demonstrate that curcumin potentiated the antitumor effects of 5-FU in the parental as well as the 5-FU resistant derivatives of these cells in a three dimensional high density environment resembling the *in vivo* situation. Our presented results underline the tumor inhibiting effect of curcumin and demonstrate the potential benefit in chemotherapy through combination of curcumin and 5-FU through a synergistic action in enhancing tumor cell death also by influencing the MMR-system.

We further found that 5-FU or curcumin treatment individually, or in combination (curcumin with 5-FU), significantly suppresses CSCs as revealed by decreased expression of specific CSCs markers (CD44, CD133 and ALDH1) and colonosphere formation in MMR-deficient and proficient tumor cells and their chemo-resistant counterparts in high density cultures. Only a small subgroup of the tumor cell population is responsible for tumor invasion and renewal potential, also referred to as cancer stem cells (CSCs), which express cell surface markers such as CD44, CD166, CD133 and ALDH1 [45]. It has been reported that the lack of a three-dimensional surrounding in a monolayer culture may reduce the stem cell surface marker expression in a tumor cell culture [46], therefore in this study, we have used a high density culture 3D model, as this resembles the extensive cell–cell interactions observed *in vivo*. Further, it has been suggested that CSCs develop resistance to chemotherapeutic drugs and induce remission, providing basis for why cancer cells cannot be completely destroyed by conventional chemotherapeutic agents [14]. Interestingly, it has been previously suggested that development of tumor cell resistance to chemotherapeutic agents is correlated to an increase in cancer stem cell (CSC) population and enhanced ability to form colonospheres [24]. These observations may help explain why it is difficult to completely abolish tumor cells and why nearly 50% of patients develop tumor recurrence after few years of their cancer treatment. Several lines of evidence have suggested that many different tumor cells develop from a small subpopulation of CSCs through their oncogenic transformations [47], [48] and that, in addition, CSCs can develop from healthy and progenitor cells by transforming gene mutations, since many of them have still features from origin and surrounding differentiated cells, implicating that these mutations may happen in differentiated tissue cells [49], [50], [51].

Therefore, it is quite challenging to investigate agents that can effectively downregulate CSCs in colon cancer cells, and decrease the carcinogenic potential of CRC. Indeed, our results are consistent with studies showing that curcumin together with FOLFOX (5-FU and oxaliplatin) decreased the expression of several colon CSC receptors, EGFR and inhibition of colonosphere-formation in the FOLFOX-surviving cells [24]. Although, the molecular mechanisms that support curcumin-induced inhibition of tumor cell growth is not fully understood, the results of other investigations and our laboratory have shown that curcumin blocks the activation of β-catenin as well as NF-κB [5], [52], [53]. Furthermore, it has been discussed that increasing the expression of CSCs in FOLFOX-treated CRC cells and development of chemo-resistant potential could be induced by changes in gene-hypermethylation and stabilizing chromatin thus blocks binding of transcription factors to genes, which is downregulated by curcumin [24], [54], [55]. Indeed, Hollenbach et al. demonstrated that loss of MMR provides an *in vivo* survival advantage to the stem cell population in the presence of DNA damage that may have implications for cancerogenesis [56].

In most human cancers with DNA MMR deficiency, there is a direct correlation between a damaged cell and emergence of chemotherapy resistant CSCs [57]. It is believed that the activation of DNA MMR system would help in inhibiting the survival mechanisms by raising the sensitivity of such cells to therapeutic drugs and reduce its tumor potential by arresting their growth. Owning to the mass effect, progeny cancer cells still show the symptoms in the patients and therefore, the combinational therapy using curcumin and 5-FU for the eradication of cancer stem cells and differentiated cells would be more effective. As a result, anti-tumor treatment strategies selectively targeting the subset of tumor stem cells would be of tremendous clinical significance for the management of such patients.

In conclusion, in this report, we provide novel evidence and previously unrecognized effects of curcumin, showing that curcumin individually or in combination with the conventional colon cancer chemotherapeutic agent 5-FU could serve as an effective therapeutic strategy to prevent the emergence of chemo-resistant colon cancer cells by reducing CSCs. These results are particularly important from a clinical standpoint, where reduced toxicity towards the patients is often sought when using these toxic chemotherapeutic drugs. Our data suggest that a combination treatment of phytochemicals together with conventional chemotherapeutic agents can be very beneficial for the patients in enhancing the clinical therapy and simultaneously reducing side effects through lower dosage requirements. Furthermore, MMR-proficient HCT116+ch3 and their 5-FU resistant counterpart cell lines are more sensitive to 5-FU and/or curcumin, indicating an important role for the involvement of DNA MMR-system in mediating chemosensitization in cancer cells - a topic that is of burgeoning clinical relevance for the management of CRC patients that frequently become refractory to conventional chemotherapy.
